# Introducing a national essential diagnostics list in India

**DOI:** 10.2471/BLT.20.268037

**Published:** 2020-11-30

**Authors:** Sonam Vijay, Raman R Gangakhedkar, Chander Shekhar, Kamini Walia

**Affiliations:** a Division of Epidemiology and Communicable Diseases, Indian Council of Medical Research, Ansarinagar, New Delhi, 110029, India.; b Division of Innovation and Translational Research, Indian Council of Medical Research, New Delhi, India.

Accurate and affordable diagnostics are central to effective health care. Quality diagnostics, when made available as part of health-care services, influence disease diagnosis and treatment outcomes in patients and improve disease surveillance. Poor investment in diagnostics, especially in low- and middle-income countries, has translated into high out-of-pocket expenses on diagnostics. Furthermore, lack of diagnostics can result in serious public health challenges, such as antimicrobial resistance due to overuse of antibiotics.^1^ Lack of quality diagnosis is now being acknowledged as an important obstacle to improved health care. Recognizing that absence of quality diagnosis is an important gap in the health-care system, the World Health Organization (WHO) published the *First WHO model list of essential in vitro diagnostics* in 2018; the Indian Council of Medical Research (under the Ministry of Health and Family Welfare) followed in its footsteps and released its first *National essential diagnostics list* in 2019.^2^ India has had a national list of essential medicines for the past 20 years; however, diagnostics remained a neglected area. With the Indian government aiming to achieve universal health coverage, the diagnostics list is a breakthrough step to make reliable high-quality diagnostic testing an essential component of the health-care system, therefore providing universal access to affordable, accessible and good-quality health services to all Indian citizens.

India has had a long history of vertical, disease-specific programmes, which contributed immensely to the control of infectious diseases such as human immunodeficiency virus (HIV), tuberculosis and malaria.^3^ In the last decades, the burden of disease has gradually shifted, with noncommunicable diseases growing from 30% in 1990 to 55% in 2016.^4^ While vertical programmes were successful in improving access to treatment and diagnostics for specific diseases, the availability of diagnostics for conditions other than the ones covered in the vertical programmes is inadequate across the health system. Findings from a survey conducted in primary health centres in three Indian states in 2018 revealed gaps in the availability of essential tests for malaria, HIV, hepatitis and other diseases.^5^ Other studies reveal critical gaps in the availability of key diagnostics needed for the management of noncommunicable diseases at primary health facilities.^6^ Some primary health centres and community health centres had shortages of glucometer and electrocardiogram machines and most primary health centres lacked point-of-care test supplies such as blood glucose estimation strips, needles and urinary protein strips.^6^ To respond to the changing health-care needs of the population, deconstructing the vertical disease-specific model is necessary; doing so would create a more comprehensive and stronger horizontal programme that addresses the diagnostics needs of patients. 

India’s essential diagnostics list is comprehensive and ambitious; through this list, the Indian Council of Medical Research seeks to cement diagnostics as an important component in health care. The list should translate into improved patient outcomes and reduction in out-of-pocket expenses on diagnostics. The list was drafted through a consultative process spearheaded by the Indian Council of Medical Research. The process of reaching a consensus on which diagnostics to include in the list consisted of five consultative meetings with clinicians, microbiologists, pathologists, radiologists, representatives of civil society, managers of national programmes, representatives of the diagnostics industry and other technical experts over a period of 15 months. The consultations focused on the latest available evidence of disease burden,^4^ the changing health-care needs and the stakeholders’ expectations from the essential diagnostics list. The list includes 117 general laboratory tests for a broad range of common conditions for the diagnosis of communicable and noncommunicable diseases: the 29 disease-specific tests for HIV, hepatitis, tuberculosis, dengue, malaria and area-endemic diseases, and 24 imaging tests such as X-rays, computerized tomography scans, magnetic resonance imaging scans and ultrasound sonography. The list has been strategically harmonized with other ongoing government initiatives, such as various disease-control programmes, the Indian Public Health Standards and the Free Diagnostics Service Initiative to ensure that the diagnostics list is supported by all initiatives.^2^

In 2015, the Indian government, under the guidance of the National Health Mission, launched the Free Drugs Service and Free Diagnostics Service Initiative, as part of its nationwide effort towards the government-led Health for All objective.^7^ The Initiative, aimed at reducing out-of-pocket expenses on diagnostics by providing free quality diagnostics services at all levels of health care, has adopted the recommendations of the diagnostics list. Of the overall out-of-pocket expenses on health care, the current expenses of Indian patients on diagnostics is estimated to be around 10%.^8^ The Initiative and the states make annual investments into the service of about 175 million United States dollars to meet the costs of conducting tests, maintaining equipment and consumables.^7^ The initiative, aware of the deficient laboratory systems and how these deficiencies may impact implementation of the diagnostics list, has released a guidance document to assist the states in strengthening health-care systems to deliver diagnostics services.^9^ The document provides guidance on how to analyse gaps in existing equipment availability, procurement, management of supply chain, human resource hiring and training, quality control and data management and analysis. The Initiative is encouraging states to create systems to deliver diagnostics using public–private partnerships, strengthening in-house capacities, or using private providers when required. The initiative is being implemented in 24 states through different models. Many states have adapted the suggested solutions to their local context and are successfully implementing the Initiative, indicating that the proposed mechanisms will be helpful in overcoming the challenges of implementing the diagnostics list. With the current inadequate resources and infrastructure, encouraging public–private partnerships seems a rational decision; however, these partnerships should only be seen as a transitory arrangement. The overall aim should be to create structures that are sustained and supported by the government. The Initiative is completely funded by the Government of India and has a separate earmarked budget for diagnostics, showing that the government acknowledges the importance of diagnostics as an indispensable component of the health-care system, and forecasting the Initiative’s sustainability.

Having a national essential diagnostics list endorsed by all major stakeholders would have several advantages, mainly homogenizing diagnostics availability at every level. States can use the list as a reference document to forecast needs and guide their procurement; the list can also help them determine the volumes of tests and mechanisms required to meet demand and negotiate diagnostics’ prices. Special pricing initiatives driven by aggregated volumes have been practiced by some disease control programmes in low- and middle-income countries to improve diagnostics availability.^10^ The list is also an opportunity for the industry to focus its resources on the diagnostics that the government is expected to procure, thus contributing to avoiding shortages and maintaining supply chains. The list can also help simplify the regulators’ task of stopping suboptimal diagnostics, such as the tuberculosis serodiagnostic test kits, which were common in a climate of poor regulatory set-up and misguided tuberculosis diagnosis in India.^11^

During the consensus-building process to develop the list, the committee of experts identified several important gaps where diagnostic solutions for priority health-care conditions specific to India are needed. In the last decade, the development of point-of-care diagnostics has advanced significantly, with scientists and entrepreneurs making strides in the development of next-generation point-of-care diagnostic devices that are based on modern technologies such as smartphones and cloud-based devices. While some point-of-care diagnostics such as rapid blood glucose, digital haemoglobinometer and cardiac biomarkers have already been included in the diagnostics list, point-of-care rapid tests for the detection of infectious diseases, such as typhoid, are needed.^12^ Having point-of-care diagnostics could also benefit primary health centres and rural settings where auxiliary nurse midwives and accredited social health workers provide diagnostic services. The Indian Council of Medical Research needs to create a pathway to evaluate available point-of-care diagnostics for their uptake in the diagnostics list. To harness the full potential of the evolving diagnostics landscape, a separate Diagnostics Working Group should develop a national strategic plan focusing on research and policy on diagnostics. Such a working group could facilitate the creation of regulatory frameworks for diagnostics and laboratories; provide a pathway for the evaluation of available diagnostics for their uptake in the health-care system; and formulate a process for periodic updating of the list. These three tasks would ensure an optimal use of existing diagnostics as well as timely uptake and use of innovations in diagnostics. 

Through the national essential diagnostics list, the Indian government seeks to make the availability of quality diagnostics an essential component of the health-care system; the list can also become a guidance document for other countries to develop a similar tool. Strengthening of diagnostics infrastructure and improving the availability of diagnostics will be key to achieving diagnostics availability across all levels of health care. Even the point-of-care diagnostics, despite their promise of simplicity, affordability and shorter turnaround time, require a functional delivery system that is supported by laboratory and clinical expertise. We hope that implementing the national essential diagnostics list, which is underway through the Free Drugs Service and Free Diagnostics Service Initiative, will increase the availability of good quality diagnostics and therefore reduce out-of-pocket expenses on diagnostics and improve patient outcomes. 
